# From Individuals to Systems and Contributions to Creations: Novel Framework for Mapping the Efforts of Individuals by Convening The Center of Health and Health Care

**DOI:** 10.2196/39339

**Published:** 2022-11-03

**Authors:** Dana Lewis, Liz Salmi, Alicia Staley, John Harlow

**Affiliations:** 1 OpenAPS Seattle, WA United States; 2 OpenNotes Beth Israel Deaconess Medical Center Boston, MA United States; 3 Medidata Boston, MA United States; 4 School for the Future of Innovation in Society Arizona State University Hinesburg, VT United States

**Keywords:** patient-centered care, patient role, patient involvement, access to care, patient-centered outcomes, co-design, participatory design, patient and public involvement

## Abstract

**Background:**

People with lived health care experiences (often referred to as “patients”) are increasingly contributing to health care and are most effective when they are involved as partners who can contribute complementary knowledge alongside other stakeholders in health care.

**Objective:**

Convening The Center aimed to bring together “people known as patients”—the center of health care—to address priorities as they defined them.

**Methods:**

According to the original project design, an in-person gathering was to be conducted; however, as a result of the COVID-19 pandemic, the in-person gathering was transformed into a series of digital gatherings, including an in-depth interview phase, small-group gatherings, and a collective convening of 25 participants (22 women and 3 men from the United States, India, Costa Rica, Sweden, and Pakistan). Each participant was interviewed on Zoom (Zoom Video Communications Inc), and the interview data were thematically analyzed to design a subsequent small group and then full cohort Zoom sessions. Visual note-taking was used to reinforce a shared understanding of each individual- and group-level conversation.

**Results:**

The interviews and gatherings for Convening The Center offered unique perspectives on patient activities in research, health innovation, and problem-solving. This project further developed a novel, two-spectrum framework for assessing different experiences that patients may have or seek to gain, based on what patients actually do, and different levels of patients’ involvement, ranging from individual to community to systemic involvement.

**Conclusions:**

The descriptors of patients in academic literature typically focus on what health care providers think patients “are” rather than on what patients “do.” The primary result of this project is a framework for mapping what patients “do” and “where” they do their work along two spectra: from creating their own projects to contributing to work initiated by others and from working at levels ranging from individual to community to systems. A better understanding of these spectra may enable researchers to more effectively engage and leverage patient expertise in health care research and innovation.

## Introduction

People with lived health care experiences (often referred to as “patients”) are increasingly contributing to different areas of health care, including research [1,2]. This strategy of involvement is most effective when individuals with lived experiences are involved as research partners [3] and contribute complementary knowledge alongside other types of stakeholders [4], such as health care providers, purchasers, payers, policy makers, product makers, and principal investigators (PIs) [5]. However, these individuals are rarely presented with funded opportunities to directly connect with and learn from one another without the agenda-setting of sponsoring organizations [6,7]. To address this gap, the Convening The Center (CTC) project, funded by the Robert Wood Johnson Foundation, engaged 25 individuals representing diverse health conditions, geographies, and digital communities and provided resources to enable these individuals to gather and discuss the health care interests that mattered most to them—without influence from outside organizations. The needs of patient communities was the sole focus, and not an afterthought or engagement checkbox, of the CTC organizers (one of whom is a patient researcher herself and the other is a supportive collaborator). Embracing diversity improves patient outcomes [8]; however, diversity in patient participation in innovation remains a persistent challenge. Opportunities to participate in medical research and innovation mostly do not recognize conventional diversity criteria [9-12] or the talents and interests of patients; rather, patient participation in studies is often designed as a “check box” in funding proposals [13,14]. Archetypes of people with lived health care experiences have been defined in academic literature: the “difficult” patient [15], the “complex” patient [16], the “absent” patient [17], the “good” patient [18], or the “smart” patient [19,20]. These labels, not created by patients, fail to help researchers, health care practitioners, or other patients understand how to match the strengths, skills, and expertise of patients with participation opportunities.

This is the research gap that the CTC sought to better understand and explore. Many labels for types of patients exist; however, they are not used or adopted by the patients themselves, have not been collected into a cohesive map, and do not facilitate matching the skills and interests of patients with tangible opportunities for people with lived health care experiences to participate in improving the health care ecosystem at large.

The contributions of CTC are 2-fold: first, a patient-informed framework for mapping behaviors and activities for further study and exploration. The framework recognizes that the expertise patients bring ranges widely, including *creating* or initiating their own projects, communities, or solutions; *contributing* to other projects, research, or communities; articulating *individual lived experiences* (n=1) with particular health conditions; participating and engaging in *communities of patients* with differing lived experiences; and supplying expertise across multiple diagnoses, geographies, or digital communities at the *systems level*. Second, the methods used throughout CTC are novel and unique because they introduce participants to one another, establish trust, and facilitate conversations. This paper describes the methods of CTC, the range of topics that emerged from the cohort across the project’s discussions, the development of a novel framework for assessing patient experiences, and the potential applications of the framework for future use to improve the diversity of patient perspectives in research.

## Methods

### Overview

This formative, qualitative project used digital purposive sampling [21] of individuals with lived patient experiences. This involved a novel, three-phased approach: an initial phase to meet and develop relationships with individual participants, a second phase to engage small groups of participants to develop rapport within the cohort, and then a third and final phase to encourage deeper discussions.

Before phase 1, we sought to recruit a diverse selection of potential participants to CTC. The eligibility to participate in the project was broad; the project was open to anyone with new experience, a long history of working to improve health care through advocacy, innovation, design, research, or entrepreneurship, or other history of advocacy in a health-related domain. Initially, CTC was intended to be an in-person convening oriented to the priorities and interests of participants; travel costs and funding for participants’ time was outlined in the project budget, which was communicated to potential participants. This opportunity for the “periphery” of the health care space was intended to contrast with other health and health care events sponsored by the “center” of the health care space [22]—companies, insurers, provider networks, research funders, or academic societies—which naturally focus on issues of concern to those stakeholders rather than on the needs and quality of life of people with lived health care experiences. After the COVID-19 pandemic disrupted the plan for individuals to meet in person, CTC was redesigned as a digital activity. The reduction of travel and venue costs enabled an increase in participant honoraria. This change was announced to the initially nominated participants, and additional time was added to this stage of recruitment.

Participant recruitment was conducted in multiple stages to enable both self-nominations and nominations of others who may not have been aware of the potential opportunity, being outside of the one to two degree-connections of the research team.

For initial nominations and recruitment, a Google Form was created (Multimedia Appendix 1) and shared through a blog post on the PI’s blog (primarily about lived experiences with multiple chronic conditions) [23] and the investigators’ Twitter accounts [24], with requests for interested participants to further share the form with additional patient communities. To increase the research team’s ability to reach different communities, the form asked for additional communities and organizations that should be notified of the CTC program. After the first Google Form was closed, 90 nominees were contacted via email with invitations to complete a second Google Form (Multimedia Appendix 2) with additional demographics and a response to the question “What inspired you to want to make a difference in health care?”

The CTC PI (DL) and co-PI (JH) thoroughly reviewed all applications on an ongoing basis to ensure that a mix of individuals had been nominated to represent rural and urban settings; a diversity of ages, geographies, races, and ethnicities; and various gender orientations. After the application period closed, the investigators (DL and JH) applied diversity criteria to select a cohort that prioritized the participation of individuals who were: Black, Indigenous, or People of Color; women; and residents of rural areas. Diversity in the participants’ experience in research was also considered. A total of 41 individuals completed the second stage of nomination, and ultimately, 25 individuals were selected to participate. This cohort size was partly driven by the original budget and plans for an in-person gathering, where up to 30 US-based individuals would have been selected to participate in person. Cohort size was shaped by the research team’s availability for facilitation, the dynamics of group sizes in digital meetings [25], the time and resources necessary to support visual note-taking, and investigator assessment of how individuals were positioned to uniquely contribute to and benefit from participation in the cohort.

The 25 individuals were chosen for the cohort primarily based on their responses to open-ended questions about their work or interests in the health spaces that led them to apply for CTC. However, after the initial selection, based on the open-ended answer content, the research team reviewed additional metrics to ensure that they did not repeat the structural biases that may have influenced how individuals responded to the questions in the application for nomination. The final cohort was consequently diverse across several metrics. We asked nominees how long they had been advocating in the space they had described in their application: 8% (2/25) reported 1 to 2 years, 20% (5/25) reported 3 to 5 years, 40% (10/25) reported >5 years, and 32% (8/25) chose the option of “It’s complicated to answer - I’ve been working on multiple problems over time.” When asked for information on race, 64% (16/25) reported White, 20% (5/25) reported Asian, 8% (2/25) reported Black or African American, 4% (1/25) reported Hispanic or Latino, and 4% (1/25) reported American Indian or Latina. Age was the most balanced metric out of those that we evaluated: 20% (5/25) were aged 25 to 34 years, 28% (7/25) were aged 35 to 44 years, 24% (6/25) were aged 45 to 54 years, 20% (5/25) were aged 55 to 64 years, and 8% (2/25) were aged >65 years. Gender was the most imbalanced metric in the final cohort: 88% (22/25) of participants were women. After a deep discussion evaluating additional individuals who had identified as men based on the fit with the rest of the cohort, we ultimately did not expand the cohort to additional men participants based on gender imbalance, as we weighted lived experience higher as a criterion than attempting to increase men in the cohort. The cohort represented lived experiences across numerous areas, such as disease-specific communities (eg, lung cancer, breast cancer, diabetes, and various rare diseases), as well as cross-community topics (eg, trauma resulting from or related to health care and a focus on diversity, equity, and inclusion in advocacy spaces). The cohort was primarily based in the United States (19/25, 76%) but also included participants from Costa Rica (1/25, 4%), Sweden (1/25, 4%), India (3/25, 12%), and Pakistan (1/25, 4%).

To begin building rapport with the selected cohort, the investigators designed an informal, semistructured [26] interview protocol (Textbox 1). The goal of these conversations was to listen deeply and understand the perspectives of each participant, including changes in their efforts during the COVID-19 pandemic. The investigators interviewed each of the 25 CTC participants one-on-one through Zoom, which constitutes phase 1.

Two notetakers were present during the phase 1 Zoom calls. The first was the PI (DL). The second was RR, who kept their camera and audio off throughout phase 1 and was introduced as an additional notetaker. The lead interviewer during phase 1 was co-PI JH. This was intentional, as PI DL had existing relationships with a few participants; others may have had name recognition or awareness of PI DL’s own work in this space, and it was possible that these factors may influence discussions. PI DL self-assigned herself to a note-taking role to minimize her influence on the direction of these initial conversations. At the end of the call, PI DL was invited by co-PI JH to re-enter the conversation to help answer questions from the participants about the next steps for the project and what to expect.

Unbeknownst to each interviewee, the second notetaker during phase 1 (RR) was an artist assigned to develop a “visual note”—an illustration—of each participant’s conversation. The research team used follow-up calls to present each participant their visual illustration, which was intended to be a gift to the participants that they could use in the future. During this second Zoom call, the research team presented the artwork, sought initial reactions to it, and asked the participants to request changes or edits to the visual notes to ensure that their gift accurately portrayed their involvement and experience in addition to how they preferred the art piece to appear. This helped the research team represent participant experiences as intended and aligned the artwork with the purposes for which the participants might use it.

**Semistructured interview questions used in Convening The Center.**

**Intent and the corresponding interview questions**

- General introduction questions and seeking to understand their efforts and problem spaces
  How are you doing?
  In general, how comfortable are you talking with others about your personal health or your personal story?
  What did you have to do to prep for this call?
  Tell me about yourself and how you found yourself working to fix something in health care.
  How long have you been in the health care–fixing space?
- Addressing the elephant in the room: the pandemic
  Tell me about your biggest consistent challenges during the pandemic.
  If you transport yourself back to 2019, in the fall, before the pandemic, can you remember what your biggest challenges were then?
  Are there any pandemic-driven changes that you appreciate? What pandemic-driven change has been the best for you?
  What do you predict the biggest challenges will be in your space after the pandemic?
- Discussing any differences in how participants and their works are seen from different perspectives
  How do you think that others (physicians, friends, family, and public) see you?
  How would they describe you or your work?
  How would you like others to see you?
  How do you see yourself?
  Are you familiar with the term “imposter syndrome”? Have you ever experienced imposter syndrome? Tell me about how you felt and why you felt so.
- Learning about the types of activities
  Where do you feel like most of your patient-or caregiver-or advocacy-related time is spent?
  What type of activities do you find yourself doing most?
  Where would you like to see most of your time spent in the future?
- Learning about the skills they use and would recommend to others
  What is the most important skill set for a new patient advocate to have? Why that one?
  What one skill set do you have that you would give to a new fellow advocate or doer?
- Systems-level questions about skills and efforts that might translate to different communities
  Of your work in this space, what do you think might translate to other patient communities or other health care spaces? Why?
  Which work would not translate? Why?
  What have you absorbed or translated from another patient community or space that you have found useful?
- Learning about the ideal design of digital gathering and to inform the design of phases 2 and 3 within the project
  Tell me about the worst digital event or experience you have had during the pandemic.
  Tell me about the best digital event you have attended during the pandemic.
  Tell me about the best digital community you have been part of.

The goals of providing visual notes to the CTC participants as part of phase 1 were to achieve the following:

1. “Surprise and delight” [27] participants and signal the intent that CTC was an experience beyond what patient advocates may have come to expect from the research participation process
2. Demonstrate the commitment of material resources made to the cohort, who are often not resourced for their work
3. Show that the research team listened to and heard each participant’s individual perspectives
4. Visualize each participant’s story as an artifact under their control and with probable personal and professional applications (eg, conference talk introductions or sharing the CTC experience with family members)

After phase 1, CTC participants were invited through email to join a workspace on Slack (Slack Technologies), a digital collaboration and chat platform. Although participation on Slack was not mandatory, many joined the platform to meet one another, and many participants shared their visual notes to introduce themselves.

To prepare for phase 2, the investigators thematically analyzed the collected interview data [28]. Phase 2 consisted of 4 small-group Zoom meetings, with up to 8 participants. Groups were selected based on a mix of availability and personality to ensure to the best of the research team’s understanding of personalities that each phase 2 conversation would allow space for all voices to contribute.

Unlike phase 1—for which there was an interview protocol—no formal agenda was set for the phase 2 calls, which was core to the project’s purpose of bringing individual patients together without an agenda. To help put each group at ease, a Google Slides deck was created to visually anchor conversations, and a link to editable slides was shared with the group during Zoom calls (Multimedia Appendix 3). The slide deck included introductory (icebreaker) activities [29] to help participants introduce themselves and their work and highlighted shared interests and themes that participants might discuss, as well as introduced them to the tool used for group note-taking. Visual note-taking took place for all 4 groups during phase 2, and participants were informed that it would help summarize the group discussions. Similar to phase 1, after each session, the visual note was presented back to the group, and participants were invited to suggest edits or changes to the visual note reflecting the conversation in an attempt to continue to promote a shared understanding of the experience.

After phase 2, the research team began mapping the participants to better understand the emerging perspectives among the cohort. The perspective maps were plotted on a 2D scale, which the team called the Two-Spectrum Assessment of Patient Experience (further discussed in the Results section). From this visual plot of participants, the research team identified groupings of participants with similar experiences within the cohort, and these groupings were later used to determine the makeup of smaller groups during breakout discussions in phase 3. Using Slack, participants were surveyed about phase 3 discussion topics emerging from phases 1 and 2.

In phase 3, all 25 CTC participants gathered for a 2-hour Zoom call. Similar to phase 2, Google Slides was used to facilitate icebreaker activities as participants joined the call (Multimedia Appendix 4). After the icebreaker activities, 3 rounds of breakout discussions took place: (1) introductions, (2) affinity groups, and (3) topic-based groups. These groups have been described in detail in Textbox 2.

After the third breakout session, there was a short break, and then all the participants returned to the main Zoom room where an agenda-less discussion took place among all 25 participants. Following phase 3, the final visual note reflecting this penultimate cohort conversation was shared, and the participants were again encouraged to provide feedback, including suggested edits or changes to further adapt the visual note.

To summarize these methods, phase 1 participants were individually engaged to get acquainted with them at an individual level and develop rapport with the research team, with the goal to surprise and delight participants through their participation in this project. As for phase 2 participants, we sought to develop rapport within small groups in the overall cohort and begin to foster ideas for the subsequent agenda-less conversation. Finally, in phase 3, we intended to design a meaningful agenda-less gathering in the spirit of the original project design while providing enough structure and support to catalyze the conversation among participants. Visual note-taking was used to advance a shared understanding of the discussions at the individual and group levels for each set of conversations.

**Groups of breakout discussions in phase 3 of Convening The Center.**

**Breakout groups**

1. Introductions
  The goal of the first breakout group was to introduce the participants to fellow participants whom they had not met in the previous phase 2 small-group interactions or had not previously known outside of Convening The Center. This was an opportunity for the participants to get to know the other members of the cohort better, and there was no formal agenda.
2. Affinity groups
  The second breakout was designed around affinity groups of participants who were working at similar levels (eg, individual solutions vs community), had similar experiences (eg, working in breast cancer communities), or had expressed interest in similar future directions (eg, patient-led research). These small groupings were determined by the research team when reviewing the visual plotting of participants on the Two-Spectrum Assessment of Patient Experience and chosen following phase 2 discussions. The small groupings included participants who were newer or getting restarted, those experienced at the community level regardless of topic or space, those who were creators or initiators of projects and communities, and those with experience at the systems level across multiple communities.
3. Topic-based groups
  The third round of breakouts was based on topics that participants had identified and voted on in Slack or by email after phase 2. In this grouping, individuals joined breakout rooms based on the topics that were most interesting to them.

### Patient and Public Involvement

Patients and the public were involved with CTC at every stage, starting from its inception. This was a patient-led (DL) project, and a compensated advisory group of 2 experienced patient advocates (LS and AS) also contributed to the overall project design and recruitment strategy and helped guide the project’s development.

All participants were paid US $1000 for their time spent on participating in all 3 phases of the project: 6 hours of synchronous gatherings between phase 1 (90-minute initial conversation and 30-minute follow-up conversations), phase 2 (2 h), and phase 3 (2 h). Participants were offered the opportunity to donate their compensation to a nonprofit organization of their choice or be paid directly for their time. For some of the cohort, this was the first time that they were offered any form of compensation for their contributions as patient advocates.

Participants were given opportunities to provide feedback on the project throughout the 3 phases, including feedback on their individual artwork, preferences for the design of digital gatherings (phase 1), group visual note feedback (phase 2), and topics for breakout groups (phase 3). Investigators asked for and received feedback from the participants immediately following the project, as well as on the draft report to the funder Robert Wood Johnson Foundation, resulting in additional content for the report [30]. Similarly, this manuscript was presented in a draft form to the cohort, seeking voluntary feedback and input from the participants who chose to review it, and their input was included in this manuscript. CTC participants are recognized in the Acknowledgments section of this paper.

### Ethical Considerations

Because this project was designed to be a participatory project and not “research,” human participant approval or ethics approval was not sought in accordance with the 2018 Common Rule 45 CFR §46.102 (l)(1) of the US Department of Health and Human Services, Office for Human Research Protections [31]. The process of consent was opt-in to participate in the project at each phase, which included disabled camera and audio until a participant chose to enable (turn on) audio or video based on their individual choice. Participants were encouraged and reminded that they could turn off their video after they turned it on (if they had turned it on previously) for any reason. Once the investigator team realized that there was content worth sharing outside the project about the methods of the project and the high-level discussion themes raised by the participants, the idea of generating a paper was brought to notice of the CTC participants. As described above, the participants were invited to participate, and many provided direct input to this article. The original intent of the paper was to present the artifact of the Two-Spectrum Assessment of Patient Experiences, which has been described in further detail below and expanded upon reviewer feedback to also include elements of the novel design methods used to design the project itself.

## Results

### Overview

CTC was not conceptualized, developed, or implemented with the intention to make it an academic research project; rather, it was designed as an opportunity for people with lived health experiences (patients) to produce relevant knowledge about how patients participate in health care research and innovation. This paper reports on “patient knowledge,” which has the potential to frame research activities to better match opportunities with the individuals who would excel at them, and offers a summary of the thematic discussion topics that arose when CTC participants were encouraged to gather and set their own agenda.

### Two-Spectrum Assessment of Patient Experience

**Figure 1:**
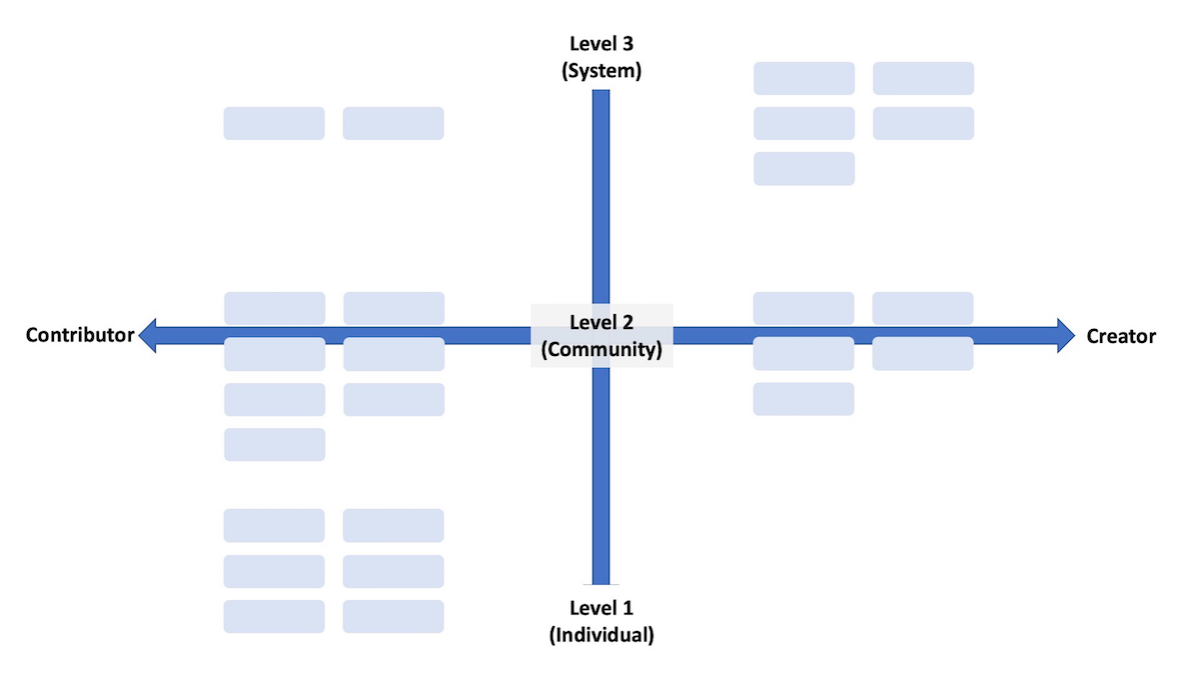
An example of the two-spectrum patient experience grid, with 25 individuals from Convening The Center mapped on the grid as assessed by the project investigators.

The primary research result from CTC was the development of a *Two-Spectrum Assessment of Patient Experience* (Figure 1).

The horizontal spectrum represents the *types* of involvement: whether someone is typically participating as a *contributor* to another project or effort, led by someone else, or are serving as a *creator* of the project or community themselves. The horizontal spectrum is “contributor” to “creator.” The vertical spectrum represents *the scale* of involvement. Level 1 indicates an *individual* level of involvement. Individuals typically start here as patients, where they identify problems or opportunities to improve things for their personal journey as a patient. Level 2 indicates a community level of involvement, that is, involvement in a community of any size, such as a disease-based community (eg, diabetes community), a specific geographic community (eg, rural Appalachia patients), or a digital community (eg, patient-run support group on Facebook). Level 3 indicates a systems level of involvement, meaning that the work transcends multiple specific communities and likely impacts multiple communities, disease spaces, or areas of health care (eg, working to improve access to electronic health records).

**Figure 2:**
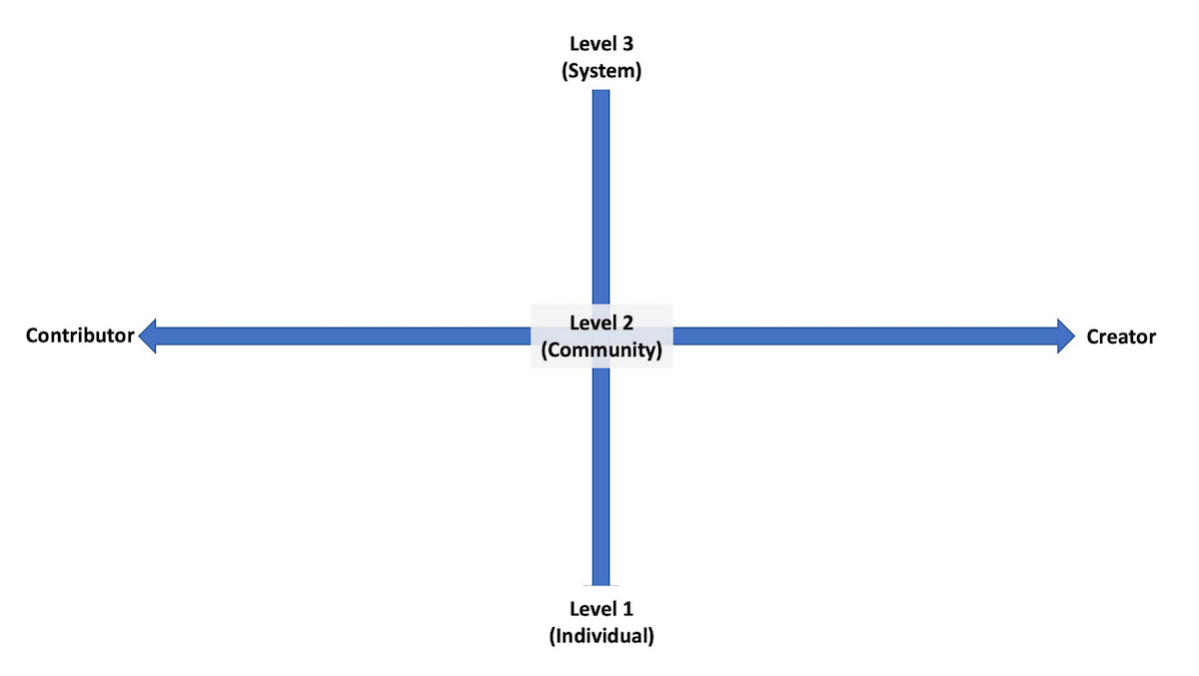
Illustrates a blank version of the two-spectrum assessment of patient experience grid as used within the project, with boxes representing individual participants based on researcher assessments of the participants’ articulation of their work during phase 1 interviews.

Figure 1 illustrates this grid as used within the project, with boxes representing individual participants based on researcher assessments of the participants’ articulation of their work during phase 1 interviews. (Figure 2 contains a blank grid for visualizing how this might be used by others in the future).

### Recurring Themes of Discussion by Participants

During phase 2 small-group discussions, some themes reoccurred independently across the groups and were voted on to be included in phase 3: research, identity, medical education and working with health care providers, and mental health.

In the resulting phase 3 discussions, the topic of *research* focused on the opportunities and experiences of patient researchers themselves (eg, to become a working member of a research team or taking on a leadership role such as a coinvestigator). These discussions covered self-directed research, and the research participants contributed to and highlighted research dissemination strategies, such as through traditional medical journals and social media.

The discussion on *identity* centered on the perspectives of being a “patient” or “carer” and identity labels (eg, diagnosis, gender, and race). Identity labels were discussed in the context of how they influence access to and care received in institutional settings, as well as how perceptions of identity can influence one’s ability to facilitate change in health care.

*Medical education* was an approach that some participants discussed as a strategy for improving the pipeline of future health care providers in training, in addition to working with and educating existing health care providers, by involving patients or carers in continuing medical education activities.

The topic of *mental health* was covered through a wide-ranging discussion about the mental health needs of individuals living with chronic illnesses, the influence of the COVID-19 pandemic on mental health overall, and the routine challenges and mental health needs that are outcomes of the health care system not serving patients well.

Additional breakout group discussions included a range of topics, some of which overlapped with phase 2 discussions, as follows:

1. The challenges of sharing patient data and experiences back with the health care system
2. Duplication of efforts across patient communities or initiatives (ie, “recreating the wheel”)
3. The unique challenges and needs around transitioning out of pediatric care as a young adult
4. The differences in advocacy roles for awareness and education compared with direct involvement in research
5. The importance of recognizing that not all patients are hyperengaged in the health care system, not all patients are seeking ways to include more stories and diverse voices, and not all patients are contributing to status quo bias by systematically excluding perspectives from harder-to-reach communities or individuals

**Figure 3:**
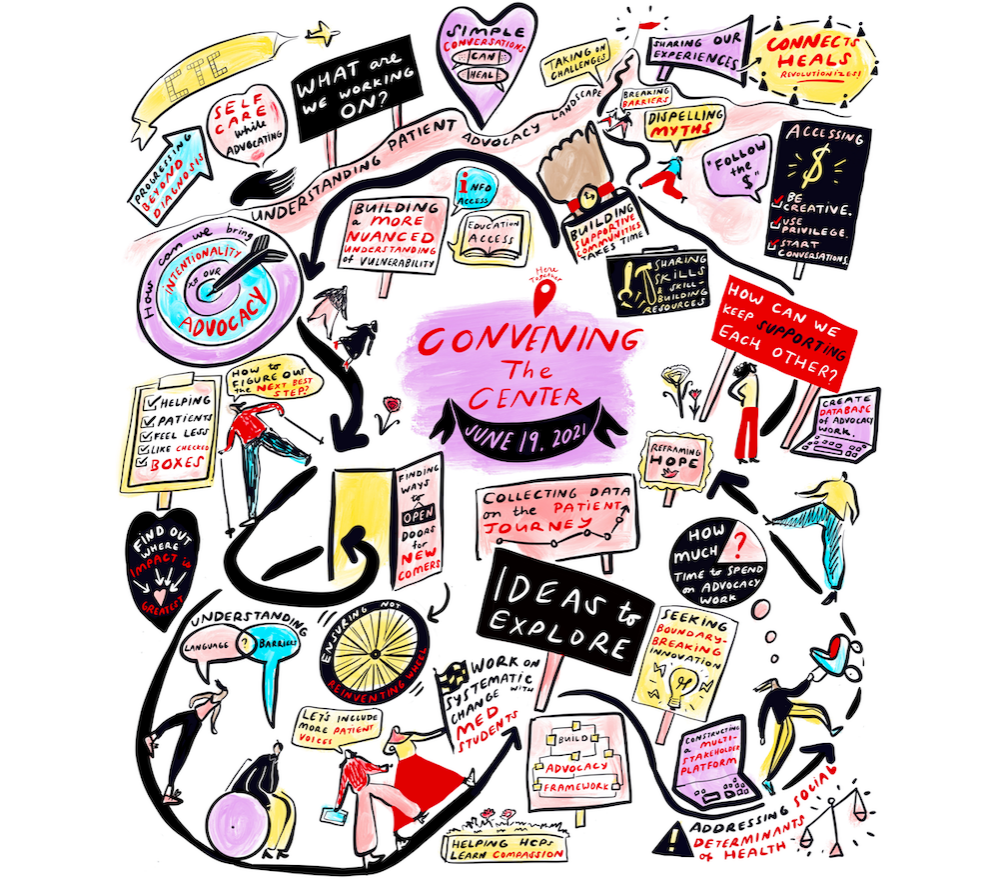
The visual note summarizing discussions in phase 3 of Convening The Center. © Rebeka Ryvola

The content discussed in phase 3 was reflected in a final visual note (Figure 3).

## Discussion

### Principal Findings

This novel, patient-led project sought to fund a gathering of diverse individuals seeking to improve health care outside of the traditional avenues of working professionally in legacy health care institutions. As a result of the COVID-19 pandemic, CTC pivoted focus to a digital series of synchronous and asynchronous gatherings to achieve the goal of bringing together a diverse cohort of patient advocates and fostering a sense of trust, openness, and community to facilitate discussions based on the interests of the cohort. As a mechanism for grouping smaller conversations within the cohort, a two-spectrum framework of patient experience was developed to articulate differences and similarities outside of typical criteria such as gender, race, geography, or disease and provide opportunities to reflect on the contributions to or creations of existing or novel efforts as well as individual-, community-, and systems-level experiences. This two-spectrum framework does not define patients as “good” or “difficult”—as seen in the academic literature sometimes—nor does it categorize patients along demographic criteria such as race, gender, geography, or disease. *Rather than making claims about what patients “are,” this framework describes what patients “do,” the often-unseen work of patients, and, importantly, how they do this work.* By better understanding and mapping what patients “do,” there is a great potential to ripple outward from the center of health care (patients) and influence future innovation throughout health care spaces.

This framework was developed to aid the research team in understanding the clusters of alignment and differences within the CTC’s 25-member cohort; however, we realized that this framework could also be useful outside of this project. First, patients may be able to use the *Two-Spectrum Assessment of The Patient Experience* grid to self-identify *where they are* currently in their experiences and interests. Second, they may be able to identify a *direction* in which they would like to head, “see” other patients in those areas, and seek mentorship, partnership, or support from those individuals. They may also use this framework to assess invitations and opportunities to contribute to traditional research projects or health care improvement initiatives.

It is important to note that not all patients intend to be involved in any particular place in this matrix. There is no right or wrong place to be or strive to be. However, the matrix can be used by individuals with lived health care experiences to self-assess their current and prospective work. It can also aid patients in assessing opportunities to determine whether they match the type of work that they want to do. Similarly, researchers, organizations, companies, and others can use this framework to better articulate and define what level of involvement and participation they are striving for when seeking patient involvement in a project or research initiative.

This framework may help all sides better articulate the expectations of what a project entails and determine whether an individual is an ideal fit for a project and importantly, whether the patient advocate is even interested in the project being presented by a funder or other entity.

For researchers, organizations, and others seeking to involve patients and members of the public in their work (eg, patient and public involvement programs), the *Two-Spectrum Assessment of Patient Experience* grid could aid in recognizing that patients are not “one size fits all.” Different patient advocates have a variety of life experiences, interests, and backgrounds from previous work, and projects may need varying levels of participation and expertise. As such, the Two-Spectrum Assessment of Patient Experience may be used to matching opportunities with the right person rather than assuming that “any patient will do” to check a box of patient involvement.

Moving forward, this framework can be used as a tool to support the increasing involvement and resourcing of patients who are seeking or find themselves facing opportunities to help fix the parts of health care that are not working for them or their communities. It can also be used to help researchers assess blind spots to identify where they may be missing additional patient expertise and potentially creating inadvertent sources of bias. It is a potential tool to help improve the relationship between traditional researchers and the invited patient contributors to research or other health care improvement initiatives (eg, the authors also built the “Opening Pathways Readiness Quiz,” intended to assess researcher readiness to collaborate thoughtfully with patients [32]). Further tools should be developed to connect patients with varied experiences and interests to research, as well as to help patients assess their fit and interest in individual opportunities.

Beyond the framework, the key themes identified in CTC that recurred across discussions of all sizes (individual, small group, and larger cohort) are worth noting for those interested in encouraging and expanding further patient involvement and engagement in research, advocacy, and other areas of health care improvement. The themes collectively reflect both avenues of opportunity—such as self-research or contributing or creating new research or contributing to medical education—as well as challenges that participants (“patients”) experience, such as grappling with their own identity or the identity of the communities in which they participate, and the mental health aspects of being involved above and beyond one’s own health care.

In addition to the recognition of the framework as a tool to assess people as multidimensional, traditional researchers should be aware that patients are unique individuals while still sometimes sharing similar challenges across different types of communities (eg, geographic or disease). Participants in CTC, especially those with a longer duration of experience, frequently remarked on observing efforts “recreating the wheel” in those newly participating. This applies to both those with lived experience as well as traditional researchers: both groups should look for examples or inspiration not only within the disease or health area on which they are focusing but also within other groups or disease spaces [33]. For example, a rare disease community may benefit from another disease community’s efforts. Traditional researchers may benefit from sharing expertise and experience for building partnerships with individuals with lived experiences. Just as cross-disciplinary collaboration in traditional research can find applications of existing solutions in new areas, cross-disease and cross-community collaborations may also inspire reuse of solutions or further innovations to solve some of the unsolved challenges that may exist in another area.

Participants in CTC expressed the desire to continue to expand their reach and impact, perhaps with new collaborations or partnerships. One idea worthy of further exploration is platforms or opportunities to “benefit all stakeholders,” which would better connect funders, traditional researchers, and organizations or institutions with patients and existing community networks. There is an awareness within this participant group of survivor bias [34] and the privileges that some participants experience or have experienced that have led to the opportunities for their contributions in these spaces. Awareness of this should also be raised and brought to the attention of traditional researchers when assessing potential partnerships with those with lived experiences.

It is worth highlighting the conscious design efforts that went into the CTC gatherings to facilitate discussions. It was not simply another digital meeting. The 3-phase design was orchestrated to bring together a group of total strangers without an agenda, which provided challenges for an in-person or digital gathering. We chose to design sessions for individuals to small groups to an entire group discussion but further broke up the all-cohort gathering into several parts to continue to grow relationships, build comfort and trust, and provide space and methods for people to communicate their ideas and interests. We invited contributions through speaking, writing through the Zoom chat functionality, and writing notes shared on Google Slides throughout the gatherings (Multimedia Appendix 4). We specifically invited contributions and edits to the visual note prepared in all three phases. This contributed to a shared understanding of the content discussed, ensured topics or moments that resonated with participants were reflected in the notes or output of the discussions, and attempted to extend the partnership of the participants in the project.

For future meetings that bring together participants with lived experiences as advocates or research partners, or in combination with traditional researchers as partners, we encourage further thought and consideration of the inclusive design of digital gatherings. Although we by no means are experts, our consideration list for this meeting and future meetings involves: conscious choice of meeting platforms that work for all participants; flexibility in whether video is “required,” for both internet bandwidth and participant energy bandwidth purposes; including breaks and being cognizant of the length of gatherings; and designing input methods to consider personality types (eg, introverts vs extroverts or different communication styles) or those who prefer written over verbal communication.

Visual note-taking played a more impactful role than expected for the research team, in addition to meeting our intended goal of “surprising and delighting” participants by gifting them with the work of art to reflect their stories and experiences as shared with us in phase 1. Visual note-taking and our methods of asking participants for edits or changes at each phase advanced shared understanding not only for the participants but also for us as organizers and researchers. The visual notetaker did not have any biases or experiences that the investigators (DL and JH) had in health care and lived experience spaces, providing a fresh perspective and neutral “ears” to each conversation, which contributed additional findings to the research team after reflecting on each discussion and each phase of the project. Using visual note-taking to support written or transcription notes for meetings or significant gatherings is a method we would recommend others use, preferably in the digital format or otherwise in a way that invites two-way contributions to highlight any missed content or opportunities to edit to ensure accuracy.

### Conclusions

CTC was a patient-led initiative funded and organized as a gathering of diverse individuals working to improve health care outside of more traditional frameworks (eg, working professionally in legacy health care institutions). Gathering digitally, the participants highlighted some of the complexities and challenges of working to change health and health care from the outside, while also highlighting similarities in efforts across different communities. In contrast to the academic literature labeling what patients “are,” the CTC *Two-Spectrum Assessment of Patient Experience* framework describes what patients “do” when they go beyond navigating their individual lived health care experiences and transition toward community- or systemic-level involvement. Better understanding and mapping what patients “do” has the potential to ripple outward from the center of health care (patients) and influence future innovation throughout health and health care spaces.

## Appendix

Multimedia Appendix 1 Initial nomination form for Convening The Center.

Multimedia Appendix 2 Second nomination form for Convening The Center.

Multimedia Appendix 3 Slides used in phase 2 small-group conversations for Convening The Center, including examples of interactive components for introducing the participants to editing the collaborative slide deck.

Multimedia Appendix 4 Slides used in phase 3 of Convening The Center, with examples of interactive components for introducing the participants to editing the collaborative slide deck and displaying the overall agenda and flow for the all-cohort conversation.

## Abbreviations

CTC: Convening The Center
PI: principal investigator
