# Supplementary material for: From Individuals to Systems and Contributions to Creations: Novel Framework for Mapping the Efforts of Individuals by Convening The Center of Health and Health Care
Source: J Particip Med. 2022 Nov 3;14(1):e39339. doi: 10.2196/39339 (PMC9672994; doi:10.2196/39339)
Supplement: Multimedia Appendix 1 [file jopm_v14i1e39339_app1.pdf]

Check out http://bit.ly/2IE0uJ3 for background on Convening The Center, and https://bit.ly/3gBAGxk for the latest plans for 2021.

The idea: What if there was a gathering for individuals working outside of traditional healthcare pathways? What if you could learn and collaborate on an emergent agenda driven by people like you?

Participants will receive an honorarium for their time and participation.

If you know of anyone (including yourself) who might want to participate in such an event, please nominate them!

* Required

1.

2.

3.

Mark only one oval.

- Myself
- Someone else *Skip to question 6*

Self-nominations are welcome!

4.

5.

Mark only one oval.

- Yes! *Skip to question 6*
- I can't think of anyone else right now. *Skip to question 9*

Fill out your nominee's information.

6.

7.

8.

Skip to question 9

Our goal is to reach a diverse network of participants. What organizations should we contact? What types of communities or particular experiences would you like to see participating?

9.

10.

Mark only one oval.

- Yes, I can introduce you.
- Sorry, I don't have a direct connection!

11.

12.

Mark only one oval.

- ( ) Yes, I can introduce you.
- ( ) Sorry, I don't have a direct connection!

This content is neither created nor endorsed by Google.

Google Forms
