# Supplementary material for: From Individuals to Systems and Contributions to Creations: Novel Framework for Mapping the Efforts of Individuals by Convening The Center of Health and Health Care
Source: J Particip Med. 2022 Nov 3;14(1):e39339. doi: 10.2196/39339 (PMC9672994; doi:10.2196/39339)
Supplement: Multimedia Appendix 2 [file jopm_v14i1e39339_app2.pdf]

Convening The Center - second phase of recruitment

Hello! If you're receiving this form, someone nominated you, or you nominated yourself, to participate in Convening The Center. At this stage, we're looking to learn more about potential participants to help us form a diverse cohort of participants (estimating that we will have about 25 total).

Please fill out this form to share more about your experiences and perspective by January 31st.

Thanks! We'll follow up in early February.

* Required

1. What is your name? *

____

2. What is your email address? *

____

3. What inspired you to want to make a difference in healthcare? You're welcome to link to a 30 second video (YouTube, Vimeo, etc.) of your answer. OR, you can write (no more than 3 sentences) below. *

____
____
____
____
____

4. How long have you been tackling this problem or this area? *

Mark only one oval.

- Less than a year, or I'm just getting started
- 1-2 years
- 3-5 years
- More than 5 years
- It's complicated to answer - I've been working on multiple problems over time

5. Have you ever felt limited in your efforts to change healthcare? If so, what limits or barriers have you faced? *

6. If you could go back in time to when you started this work, what would you tell yourself?

7. I have been to ___ health/medicine conferences

Mark only one oval.

- 0
- 1—3
- 4—5
- 5+

8. What do you find yourself repeatedly telling people who are new to this space?

____

9. What do people with more experience in this space frequently tell you?

____

10. My work in this space mostly takes place

Mark only one oval.

- On social media platform(s)
- Within an organization
- Among a small group
- Alone
- Other: _______________

11. For my work in this space, I have considered

Check all that apply.

- [ ] Starting a company
- [ ] Starting a nonprofit
- [ ] Making a product
- [ ] Hosting a conference
- [ ] Building an online community
- [ ] Building an offline community
- [ ] None of the above

Other: [ ] ____________________

12. What is your gender identity?

Mark only one oval.

- Prefer not to answer
- Non-binary
- Female
- Male
- Other: ____________________

13. What are your preferred pronouns?

Mark only one oval.

- they/them/theirs
- she/her/hers
- he/him/his
- Other: ____________________

14. What is your age?

Mark only one oval.

- 18—24
- 25—34
- 35—44
- 45—54
- 55—64
- 65+

15. What is your race?

Mark only one oval.

- Prefer not to answer
- Native Hawaiian or Pacific Islander
- American Indian or Alaskan Native
- Asian
- Black or African American
- Hispanic or Latino
- White
- Other: ______________________

16. Is there anything else you think we should know about your background, perspective, or experiences in facing problems or addressing challenges in health/care?

General logistics question about availability

Please share some basic information about your general availability preferences. If selected, we'll follow up with more details.

17. Generally speaking, does a week day, evening or weekend time work better for you for a ~2 hour call?

Check all that apply.

- [ ] Daytime during the week (M-F) is generally fine
- [ ] Evening during the week (M-F) is generally fine
- [ ] Weekend day (Saturday or Sunday) is generally fine
- [ ] Weekend evening (Saturday or Sunday) is generally fine

Other: [ ] ____________________

18. What time zone are you in?

Mark only one oval.

- ( ) Pacific
- ( ) Mountain
- ( ) Central
- ( ) Eastern
- ( ) Alaska or Hawaii
- ( ) CET or similar in Europe
- ( ) Other: ____________________

This content is neither created nor endorsed by Google.

Google Forms
