# Supplementary material for: From Individuals to Systems and Contributions to Creations: Novel Framework for Mapping the Efforts of Individuals by Convening The Center of Health and Health Care
Source: J Particip Med. 2022 Nov 3;14(1):e39339. doi: 10.2196/39339 (PMC9672994; doi:10.2196/39339)
Supplement: Multimedia Appendix 3 [file jopm_v14i1e39339_app3.pdf]

INSTRUCTIONS:

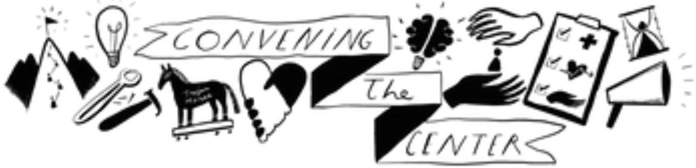

- If you’re arriving late, check the Zoom chat for which slide we are on, and jump into that activity with us.
- Feel free to explore earlier slides and add your content later!
- Please add topics, notes, and ideas for this group, or the full cohort, to the last “Parking Lot” slide in this deck.
- Questions as we go? Feel free to ask in Zoom chat! You can ask everyone, or private message John.

If you could be anywhere in the world right now, where would you be?

CONVENING The CENTER

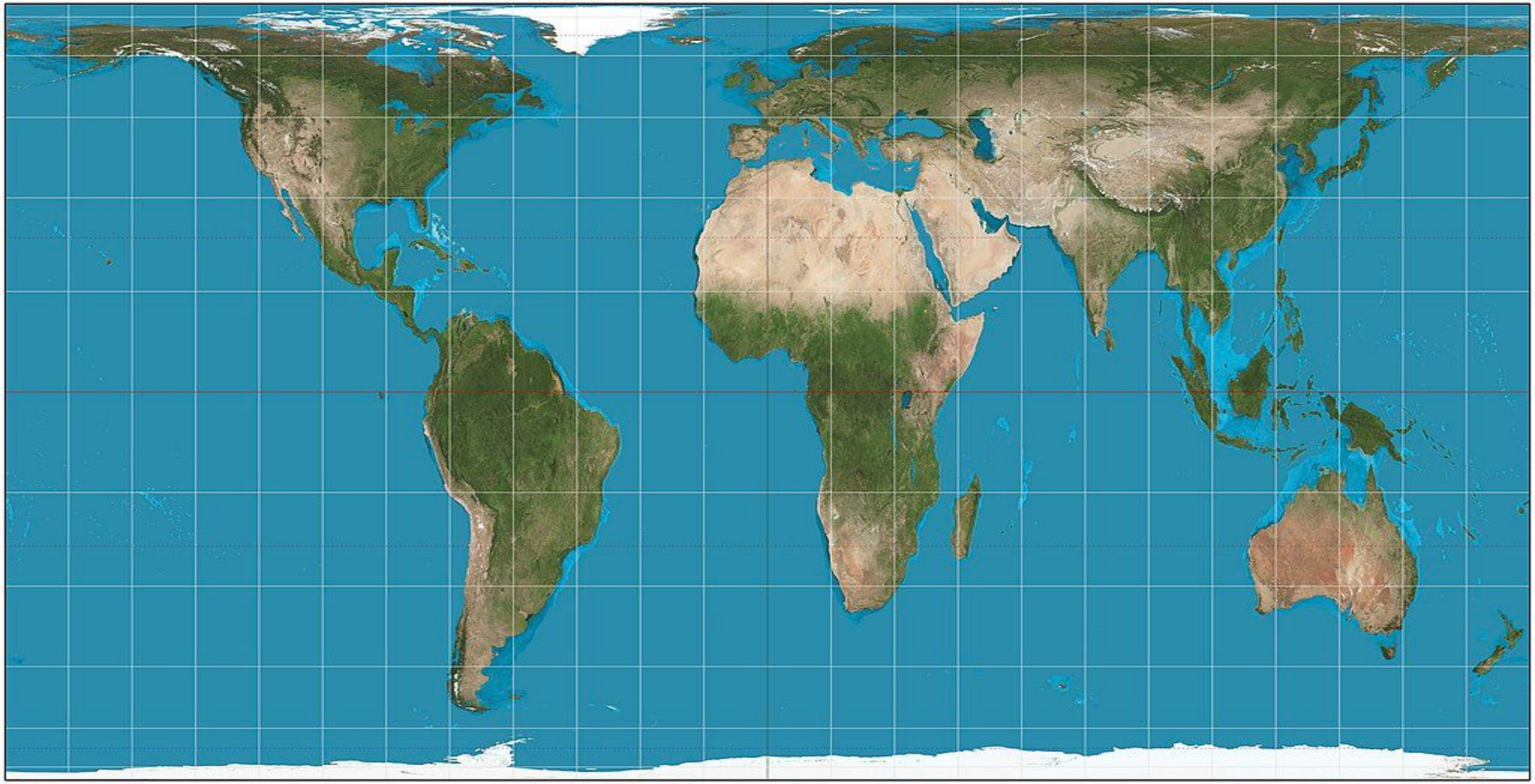

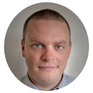

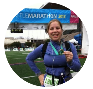

Share one “boring” or uninteresting fact about yourself:

Dana: I don’t like odd numbers!

John: My ‘environmental color palette’ is neutrals.

1:

2

3

4

5

6

7

8

Ask me about my expertise in…

(can be personal or professional)

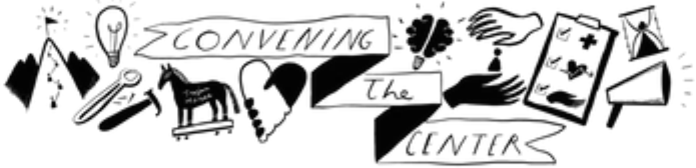

Dana: speed reading.

John: getting concert tickets for Phish.

1:

2

3

4

5

6

7

8

Where do you spend most of your time now?

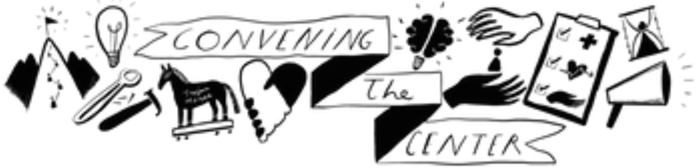

Social media and other advocacy

Research

Community support

Fundraising

Community or organizational building

I’ve been on pause

Other:

-
-
-
-
-

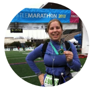

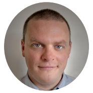

Where would you like to spend most of your time in the future?

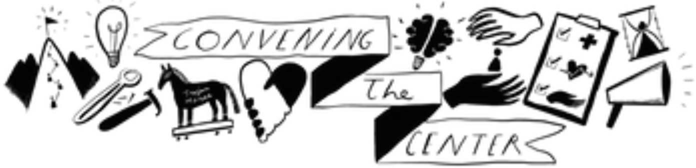

Social media and other advocacy

Research

Community support

Fundraising

Community or organizational building

Not sure

Other:

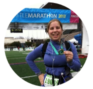

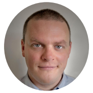

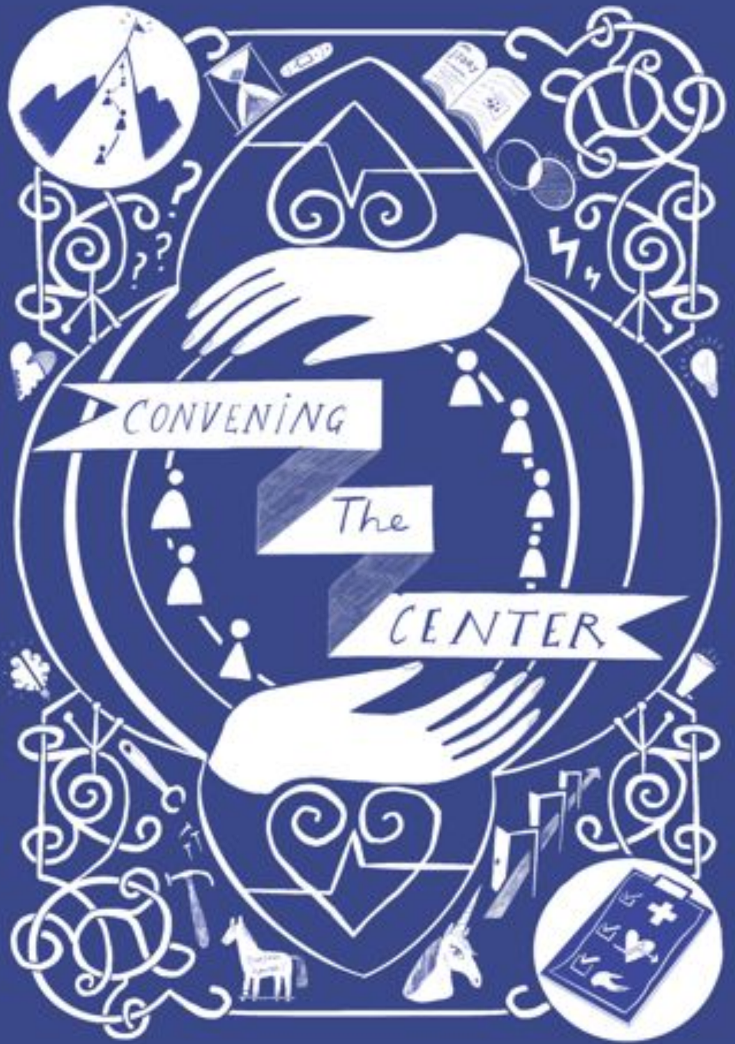

(Discussions)

How might we…. (PARKING LOT)

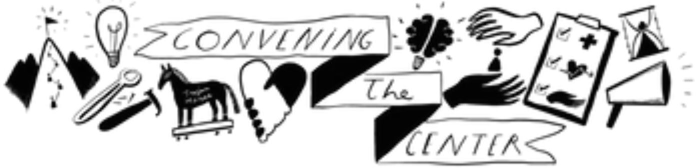

- (Leave notes here about things you'd like to bring up to discuss with your small group, either now or if we run out of time, in Slack later. Or, ideas to discuss with the entire cohort in phase 3).
-
-
-
-
-
-
-
