# Supplementary material for: From Individuals to Systems and Contributions to Creations: Novel Framework for Mapping the Efforts of Individuals by Convening The Center of Health and Health Care
Source: J Particip Med. 2022 Nov 3;14(1):e39339. doi: 10.2196/39339 (PMC9672994; doi:10.2196/39339)
Supplement: Multimedia Appendix 4 [file jopm_v14i1e39339_app4.pdf]

Find your name; click on the box and change the “fill color” of your square.

(or post in zoom chat what color you like and we’ll do it for you!)

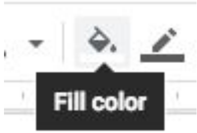

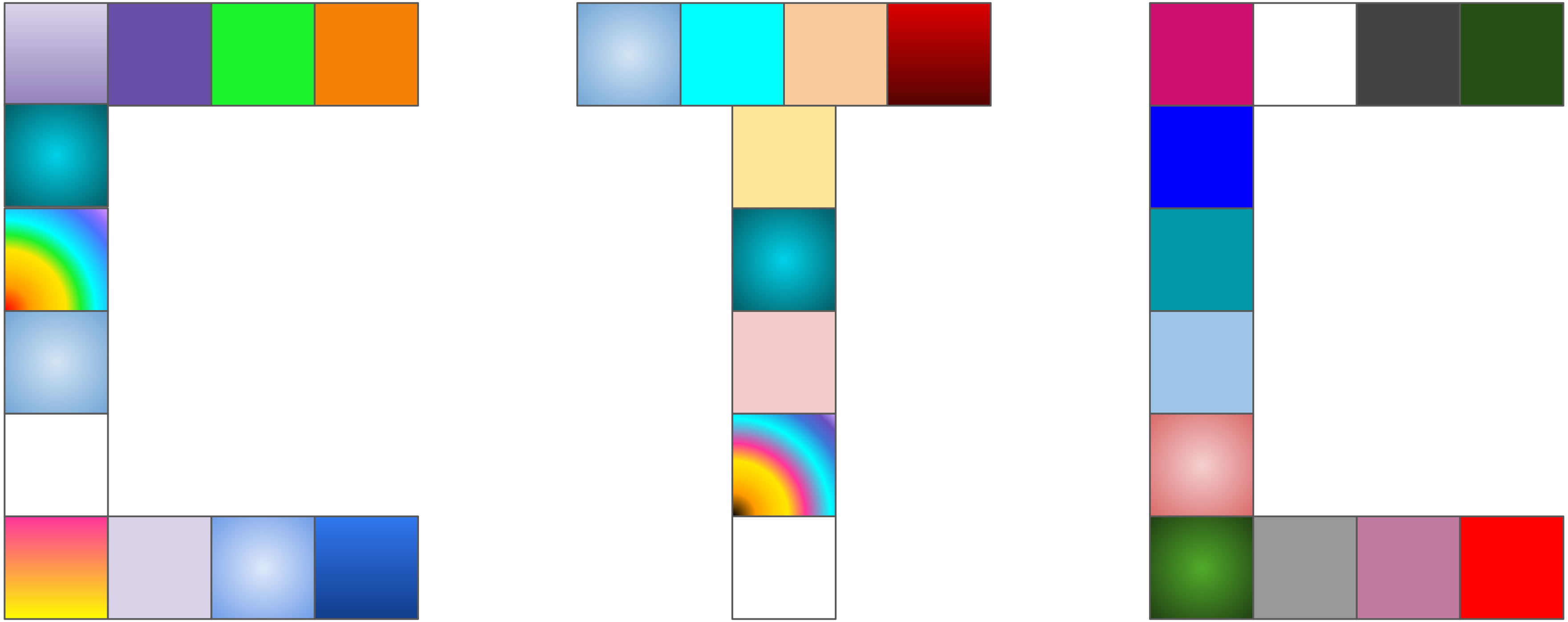

Agenda:

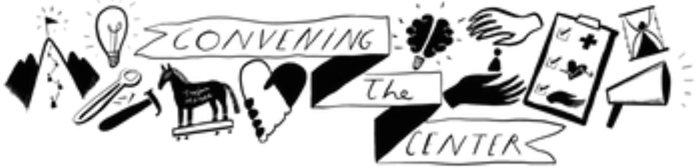

1. Overview & agenda

2. 20 minutes: Breakout Round 1 (Affinity Groups)

    Group 1: Newer or getting re-started
    Group 2: Experienced at the community level
    Group 3: Creators or initiators of projects & communities
    Group 4: Experienced at the system level (across communities)

3. 20 minutes: Breakout Round 2 (Mixing Groups)

4. 20 minutes: Breakout Round 3 (Thematic Discussions)

    - Research
    - Identity
    - MedEd/HCP
    - Mental Health

5. Everyone all together - open floor discussion
